# Searching for tunnels of proteins – comparison of approaches and available software tools

**DOI:** 10.1186/1758-2946-4-S1-P60

**Published:** 2012-05-01

**Authors:** Deepti Jaiswal, Radka Svobodová Vařeková, Crina-Maria Ionescu, David Sehnal, Jaroslav Koča

**Affiliations:** 1 National Centre for Biomolecular Research and CEITEC - Central European Institute of Technology, Masaryk University, Brno, Czech Republic, 625 00, CZ

## 

Tunnels are access paths connecting the interior of molecular systems with the surrounding environment. The presence of tunnels in proteins influences their reactivity, as they determine the nature and intensity of the interaction that these proteins can take part in. A few examples of systems whose function relies on tunnels include transmembrane proteins involved in small molecule transport and signal transduction, peptide exit channels through which ribosomes release newly synthesized proteins during transcription Knowledge of the location and characteristics of protein tunnels can find immediate applications in rational drug design, protein engineering, enzymology etc.

Identification and characterization of tunnels has been the focus of several studies, and various algorithms and software tools have been developed for these purposes [1-4]. These methodologies use special mathematical algorithms to represent and scan the surface of the protein in search for tunnels and the amino acid residues involved.

In the presented study we perform a benchmarking study of the most known approaches and software tools for finding tunnels in proteins (Mole, MolAxis, Hollow, etc.). We focused on proteins from the cytochrome P450 family, which are very important from the biological point of view. We provide a critical discussion of the strong and weak points of the analyzed approaches and software tools.
